# Negative-Pressure Pulmonary Edema Induced by Flexible Bronchoscopy: A Case Report

**DOI:** 10.7759/cureus.64352

**Published:** 2024-07-11

**Authors:** Kento Furukawa, Yuichiro Asai, Yuta Nagahisa, Keiichiro Takano, Hirofumi Chiba

**Affiliations:** 1 Department of Respiratory Medicine and Allergology, Sapporo Medical University, Sapporo, JPN

**Keywords:** negative-pressure pulmonary edema, pulmonary edema, alveolar lavage, non-cardiogenic pulmonary edema, bronchoscopy

## Abstract

Negative-pressure pulmonary edema (NPPE) arises from excessive inspiratory effort due to upper airway obstruction, often associated with postoperative laryngospasm and upper airway infections like epiglottitis. We present a case of NPPE during bronchoscopy. A 45-year-old female patient, who was undergoing bronchoscopy for interstitial pneumonia evaluation, was examined using a tracheal tube with a 7.5 mm internal diameter and a bronchoscope with a 5.9 mm external diameter. The patient's respiratory condition gradually worsened after intubation. We continued with the examination, supplying approximately 5 L/min of oxygen through the intubation tube. We performed an alveolar lavage, and the recovered fluid gradually turned pale and bloody. After the examination, the patient continued to expectorate pink and frothy sputum and prolonged respiratory failure. Chest radiography revealed new extensive bilateral infiltrates. We ruled out cardiogenic causes through clinical examination, electrocardiogram (ECG), and transthoracic echocardiography. As a result, we suspected that temporary upper airway obstruction during bronchoscopy led to NPPE. Applying continuous positive airway pressure (CPAP) quickly improved the pulmonary edema. The risk of NPPE during bronchoscopy needs to be acknowledged, especially when using larger bronchoscopes and smaller tracheal tubes.

## Introduction

Negative-pressure pulmonary edema (NPPE) is a condition in which upper airway obstruction results in excessive inspiratory effort and negative pressure in the chest cavity, leading to pulmonary edema [1]. This condition usually occurs after acute upper airway obstruction, often associated with anesthesia or postoperative laryngospasm [2]. Strong inspiratory effort against a closed glottis or obstructed airway causes increased negative intrathoracic pressure. This can lead to increased pulmonary vascular permeability and interstitial fluid accumulation. The condition typically manifests as acute respiratory distress, often accompanied by pink frothy sputum and bilateral infiltrates on chest imaging. Standard treatment includes airway clearance and positive-pressure ventilation, which is efficient [3]. However, delayed treatment of NPPE can lead to fatal outcomes [4]. There have been reports of NPPE associated with postoperative laryngospasm, but there are only a few reports on the occurrence of NPPE during bronchoscopy. Considering the pathogenesis of NPPE, it is assumed that bronchoscopy can also lead to temporary tracheal narrowing.

We report a case of NPPE that occurred during a flexible bronchoscopy procedure. The patient was a 45-year-old woman with no significant medical history who underwent bronchoalveolar lavage to investigate suspected interstitial pneumonia. Blood tests for various autoimmune markers were negative, and all hematological parameters, including coagulation factors, were within normal ranges.​​​​​​​

## Case presentation

**Figure 1:**
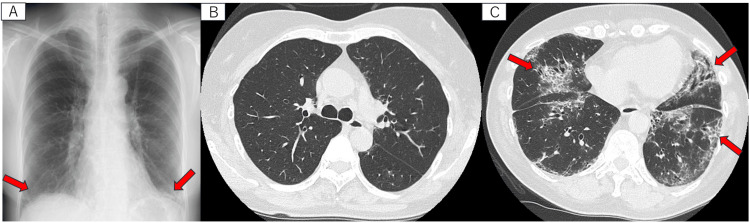
Chest X-ray and computed tomography before the bronchoscopy. There is ground-glass opacity in the bilateral lower lung fields (A). There are few lesions on the upper lobe (B). Both sides' middle and lower parts show ground-glass opacity, some with traction bronchiectasis. (C). Each red arrow indicates lesions associated with interstitial pneumonia.

A 45-year-old woman, measuring 162 cm in height and 59 kg in weight, was suspected of having interstitial pneumonia due to frost shadows observed in the lower lung fields during a routine checkup (Figure 1). To investigate further, she was admitted to the hospital for an alveolar lavage. She had no history of cardiorespiratory diseases, gastroesophageal reflux, medication use, surgeries, or allergies. Blood tests for antinuclear antibodies, anti-double-stranded DNA antibodies, myeloperoxidase-antineutrophil cytoplasmic antibodies (MPO-ANCA), proteinase-3-anti-neutrophil cytoplasmic antibodies (PR3-ANCA), and anti-glomerular basement membrane antibodies were all negative. Hematocrit and white blood cell count were within normal ranges. In addition, the platelet count and coagulation parameters, including prothrombin time and activated partial thromboplastin time, were all found to be normal. 

Before the bronchoscopy, she was administered a 15 mg intramuscular injection of pentazocine and laryngeal anesthesia with lidocaine. During the examination, she was given 3 mg of midazolam intravenously. We confirmed the patient was sedated yet breathing spontaneously and then started the examination. We used a bronchoscope (Q290 video bronchoscope; external diameter, 5.9 mm; Olympus, Tokyo, Japan) for the bronchoalveolar lavage and intubated her with a 7.5 mm inner diameter endotracheal tube (Portex® Soft Seal Cuffed Endotracheal Tube; external diameter, 10.9 mm; Smiths Medical, Minneapolis, MN, USA). The tube had a cuff, but it was not inflated with air.

The patient had a strong cough during the examination due to bronchoscopy irritation. To manage this, lidocaine was applied inside the trachea via bronchoscope (spray-as-you-go technique [5]) as needed while the examination continued. No abnormal findings were observed in the trachea, and the sputum was minimal and serous in nature. After intubation, respiratory failure occurred, necessitating an increase in oxygen supply to approximately 5 L/min. The patient's strong cough, which interfered with normal spontaneous breathing, was thought to be the cause of her respiratory failure. We administered lidocaine via bronchoscope to alleviate the cough and waited for breathing to normalize. After a while, the coughing subsided and the breathing pattern returned to normal. We decided to continue the examination while administering oxygen at 5 L/min.

**Figure 2:**
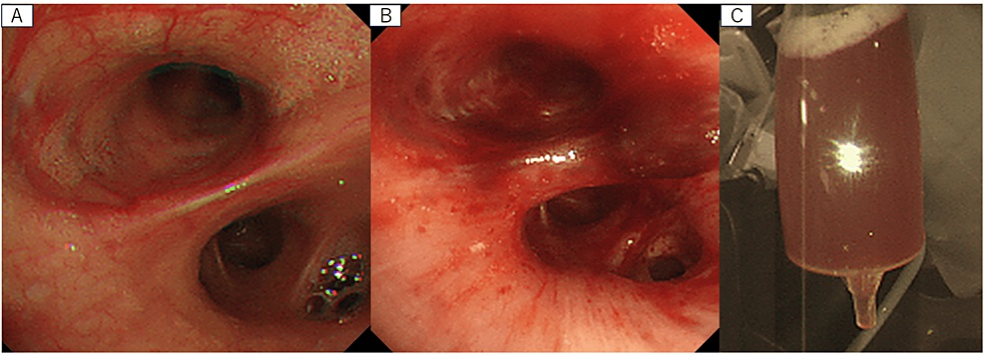
Photographs before and after alveolar lavage and alveolar lavage fluid. The right middle lobe before alveolar lavage (A). Hematogenous exudate was seen after alveolar lavage (B). Hematogenous alveolar lavage fluid (C).

The lavage was performed on the RB4. We injected 50 ml of saline aliquots using syringes, manually aspirating the fluid after each injection. This process was repeated three times. When 100 ml of saline was injected during the alveolar lavage, it appeared serous and clear. However, after administering 50 ml more of saline, the fluid became pale and bloody, as shown in Figure 2. Finally, a total of 108 ml of alveolar lavage fluid was collected.

**Figure 3:**
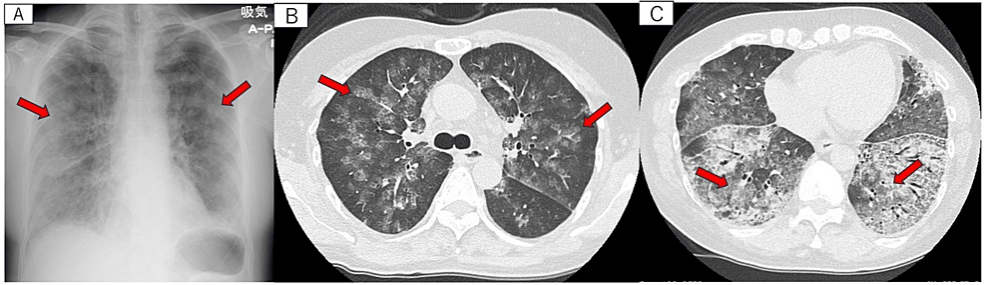
Chest X-ray and computed tomography after the bronchoscopy. Chest X-ray shows bilateral diffuse infiltrative shadows (A). The computed tomography reveals new bilateral infiltrates, primarily central in location. It also shows thickening of the interlobular septa and patchy shadows (B,C). Each red arrow indicates new ground-glass opacity.

By the end of the lavage, the patient required an oxygen supply of 10 L/min. We hypothesized that the respiratory failure was due to respiratory depression, a side effect of the sedation. Consequently, we promptly finished the examination and administered flumazenil. The patient regained consciousness and her breathing pattern returned to normal. She was able to speak normally without a hoarse voice. Her oxygen requirement was reduced to 8 L/min, but she still exhibited signs of respiratory failure. In addition, she continued to expectorate pink and frothy sputum and presented with diffuse bilateral moist rales. To identify the cause of her symptoms, we conducted a chest X-ray, which revealed extensive bilateral infiltrative shadows. The computed tomography (CT) scan revealed new bilateral infiltrates, primarily central in location, and multiple patchy densities across both lungs (Figure 3). We suspected pulmonary edema and considered the possibility of cardiogenic factors. However, echocardiography excluded primary heart issues as the cause.

**Figure 4:**
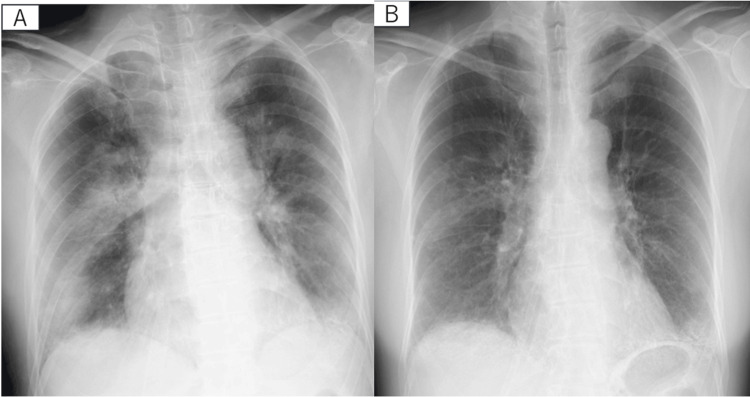
Chest X-ray one day after bronchoscopy (A) and two days after bronchoscopy (B).

Therefore, we suspected NPPE, likely caused by temporary upper airway obstruction during the bronchoscopy, and applied CPAP at 8 cmH_2_O with a fractional inspired oxygen (FiO_2_) of 0.4. This intervention rapidly improved her respiratory condition. Two hours later, we decreased the FiO_2_ in the CPAP to 0.3. Twelve hours after starting CPAP, she was able to wean off it and was transitioned to a nasal cannula delivering 2 L/min of oxygen. By the fourth day after the examination, her respiratory status had normalized. Chest X-rays showed gradual improvements (Figure 4).

The results of bronchoalveolar lavage revealed a lymphocyte-predominant cellular profile, with lymphocytes accounting for 32.0% of the cells. The distribution of other cell types was as follows: macrophages (61.0%), neutrophils (5.0%), and eosinophils (2.0%). In addition, the CD4/CD8 ratio was 2.0.

## Discussion

NPPE can occur during bronchoscopy, particularly when larger bronchoscopes and smaller tracheal tubes are used. When NPPE is suspected, it is recommended to promptly initiate positive pressure ventilation. Sharing our experience aims to improve patient safety in bronchoscopy by increasing awareness among healthcare professionals.

NPPE is a result of excessive inspiratory effort due to upper airway obstruction, which leads to pulmonary edema from negative thoracic pressure [1]. It is often associated with postoperative laryngospasm, upper respiratory tract infections, and airway obstruction from tumors [6]. Out of 74,770 bronchoscopy procedures, two cases of pulmonary edema as complications have been reported, although the specifics of these cases are not documented [7]. There is a case report that NPPE occurred during bronchoscopy [8]. In this instance, the inflammatory reaction associated with a mycoplasma infection was also considered a cause of pulmonary edema. Therefore, despite the limited number of reports, it is plausible that NPPE may occur during bronchoscopy, particularly when larger bronchoscopes and smaller tracheal tubes are used. In the aforementioned case [8], a tracheal tube with a 7.5 mm inner diameter and a bronchoscope with a 4 mm outer diameter at the tip. In our case, we used a tracheal tube with an inner diameter of 7.5 mm and a bronchoscope with an outer diameter of 5.9 mm. The patient was a petite woman, making the insertion of an 8 mm tracheal tube challenging. Therefore, we used a 7.5 mm tube. This resulted in an open but narrow lumen. Considering the external dimensions of the tracheal tube, its outer diameter is 10.2 mm. Compared to the average inner diameter of the trachea in women, which is 11.6 mm [9], this indicates a similarly narrow space. It is conceivable that such narrow spaces could have been temporarily obstructed by sputum, and furthermore, the strong inspiratory pressure associated with coughing could have led to NPPE. Men may be at relatively low risk, given that the average inner diameter of the trachea is 12.6 mm [9]. As noted in the guidelines, it is recommended that the bronchoscope be at least 2 mm larger than the tracheal tube when used [10].​​​​ ​*​ ​*

Coughing may also have contributed to the development of negative pressure pulmonary edema in the present case. During a cough, there is initially a deep inspiration. If there is any obstruction or resistance in the upper airway during the inspiratory phase of coughing, an even stronger negative intrathoracic pressure can be generated as the person attempts to inhale forcefully against this resistance. The strong negative intrathoracic pressure created during forceful inhalation against resistance can lead to NPPE.

The gradual change of sputum to a foamy pink color during bronchoalveolar lavage and even hemoptysis after the examination was completed, as in the present case, may have been caused by alveolar hemorrhage in addition to NPPE. This observation is consistent with the findings reported by Papaioannou et al. [11] who noted that hemoptysis due to NPPE, which occurs during upper airway obstruction, such as laryngospasm, may be caused by negative pressure tracheobronchial injury or alveolar capillary destruction. Indeed, alveolar hemorrhage secondary to NPPE has been reported in the literature [12], suggesting that it could be a potential complication in such cases.

Factors for differentiation in this case include laryngospasm, alveolar hemorrhage caused by vasculitis or collagen disease, and acute exacerbation in interstitial lung disease (AE-ILD). Although laryngospasm associated with bronchoscopy has been reported [13] ,it was ruled out in the present case as the patient did not exhibit inspiratory stridor as seen in laryngospasm and was able to speak. The possibility of alveolar hemorrhage secondary to vasculitis or collagen disease was considered but deemed unlikely based on negative results from comprehensive screening blood tests and physical examination. The patient was not taking any oral medications, ruling out drug-related factors. In addition, platelet count and coagulation capacity were normal before the bronchoscopy. Although aminoacyl-tRNA synthetases are positive, it is atypical that occur alveolar hemorrhage [14]. The diagnosis of AE-ILD is often difficult to confirm and must be considered after the exclusion of differential diagnoses [15]. In the current clinical setting, quickly identifying the exact condition is not critical. Immediate action is vital for life-saving interventions. When non-cardiogenic pulmonary edema is diagnosed, it is important to swiftly initiate positive pressure ventilation therapy. While treating pulmonary edema, we can simultaneously diagnose and manage the underlying cause without delay [16]. Recovery is usually rapid and within 12-48 hours with appropriate management [1]. However, mortality rates range from 11% to 40% when diagnosis and treatment are delayed [4]. In the present case, interstitial pneumonia was ruled out due to early improvement.

To prevent the occurrence of NPPE during bronchoscopy, as seen in this case, it is crucial to use larger tracheal tubes and smaller bronchoscopes. Furthermore, adequate sedation is also important to prevent coughing, which was a factor in the development of NPPE in the present case. As recommended in the guidelines [10], a combination of midazolam or propofol with an opioid is preferred over using either medication alone. In addition, the use of dextromethorphan has been reported to reduce coughing and sputum production during the procedure [17], suggesting that it could be a valuable addition to the sedation protocol. These approaches can potentially enhance patient comfort and safety during bronchoscopy.

## Conclusions

The risk of NPPE during bronchoscopy should be recognized, particularly when a larger bronchoscope and smaller tracheal tube are used. In addition, the risk is considered higher in female patients with relatively narrow tracheas. It is crucial to treat NPPE quickly to avoid fatal outcomes. When suspected, early application of positive pressure ventilation such as CPAP is essential. To prevent NPPE, it is recommended to administer adequate sedation or laryngeal anesthesia during the examination and perform sputum suctioning when necessary, in order to prevent the induction of coughing or inhalation efforts.

This case suggests that NPPE can occur in situations beyond postoperative laryngospasm and upper airway infections. Sharing this experience aims to increase awareness and preparedness among healthcare professionals, which could lead to improved patient safety and outcomes in bronchoscopy and related procedures.​​​​​​
